# Multi-omics approaches for precision obesity management: Potentials and limitations of omics in precision prevention, treatment and risk reduction of obesity

**DOI:** 10.1007/s00508-022-02146-4

**Published:** 2023-01-30

**Authors:** Selam Woldemariam, Thomas E. Dorner, Thomas Wiesinger, Katharina Viktoria Stein

**Affiliations:** 1 Karl Landsteiner Institute for Health Promotion Research, 3062 Kirchstetten, Austria; 2 Academy for Ageing Research, House of Mercy, 1160 Vienna, Austria; 3 Department of Public Health and Primary Care, Leiden University Medical Centre, 2511 DP The Hague, The Netherlands

**Keywords:** Multi-omics, Health promotion, Metabolomics, Microbiomics, Precision obesity prevention

## Abstract

**Introduction:**

Obesity is a multifactorial chronic disease that cannot be addressed by simply promoting better diets and more physical activity. To date, not a single country has successfully been able to curb the accumulating burden of obesity. One explanation for the lack of progress is that lifestyle intervention programs are traditionally implemented without a comprehensive evaluation of an individual’s diagnostic biomarkers. Evidence from genome-wide association studies highlight the importance of genetic and epigenetic factors in the development of obesity and how they in turn affect the transcriptome, metabolites, microbiomes, and proteomes.

**Objective:**

The purpose of this review is to provide an overview of the different types of omics data: genomics, epigenomics, transcriptomics, proteomics, metabolomics and illustrate how a multi-omics approach can be fundamental for the implementation of precision obesity management.

**Results:**

The different types of omics designs are grouped into two categories, the genotype approach and the phenotype approach. When applied to obesity prevention and management, each omics type could potentially help to detect specific biomarkers in people with risk profiles and guide healthcare professionals and decision makers in developing individualized treatment plans according to the needs of the individual before the onset of obesity.

**Conclusion:**

Integrating multi-omics approaches will enable a paradigm shift from the one size fits all approach towards precision obesity management, i.e. (1) precision prevention of the onset of obesity, (2) precision medicine and tailored treatment of obesity, and (3) precision risk reduction and prevention of secondary diseases related to obesity.

## Introduction

The obesity epidemic is one of the most important public health concerns in the world today. The World Health Organization (WHO) estimates that overweight affects 30–70% and obesity affects 10–30% of the adult population globally [1]. There is also a rising concern that obesity rates have tripled in the past 30 years and continue to spread in both developed and developing nations. The WHO predicts that every fifth adult in the world will suffer from obesity by 2025.

Despite the growing recognition of the disease, obesity does not receive enough attention that is proportionate to its increased prevalence and impact [2]. Evidence has shown that obesity is a risk factor for developing many noncommunicable diseases, such as diabetes mellitus, cardiovascular diseases, musculoskeletal disorders, and certain types of cancer [3, 4]. Apart from the associated health problems for individuals living with obesity, the social, medical, and economic costs can also be a burden on societies. Besides, obesity in childhood and adolescence can lead to social stigma and isolation, decreased life expectancy, a high frequency of sick leave, disability pension, and increased mortality.

To date, not a single country has successfully been able to curb the accumulating burden of obesity [5, 6]. One explanation for the lack of progress is that most approaches focus on the treatment of the medical consequences once symptoms are manifested, rather than prevention. Secondly, even those public health initiatives that are directed towards obesity prevention and management show little evidence of success and efficacy at the population level. The lack of measurable change illustrates that obesity is a multifactorial chronic disease that cannot be solved by simply promoting better diets and more physical activity.

Another apparent challenge is the traditionally simplistic calculation of severe obesity. Obesity is commonly characterized as the accumulation of body fat that results when energy intake exceeds energy expenditure, although individuals respond differently to this imbalance due to genetic predisposition. Currently, obesity diagnostics are based on standardized phenotypical body characteristic parameters: body mass index, BMI (≥ 30 kg/m^2^), body weight, waist circumference [7] and clinical parameters, such as plasma lipid profile, glycated hemoglobin, insulin, and fasting glucose levels to determine obesity comorbidities [8]. Although they are commonly used methods for assessing morbid obesity, they are limited in their estimate for risk prediction and capability to provide insights into specific molecular changes and the biochemical alterations in the development of obesity.

Hence, obesity prevention and management require a novel approach. Precision medicine promises to reform our understanding of health on the microlevel by incorporating individual characteristics such as genetics, lifestyle, and environmental risk factors. It is a personalized diagnostics approach that provides care to “the right individual at the right time” [9]. When applied to obesity prevention and management, precision medicine could bring forward knowledge on the etiology of obesity and its pathophysiological links with chronic diseases by collecting a wide range of omics data (i.e., genetics, metabolomics, and microbiome). For instance, a recent Hungarian study has highlighted the role of genetic and epigenetic factors to further understand the mechanisms involved in obesity development [10]. The study investigated and genotyped 20 single nucleotide polymorphisms (SNPs) that were believed to be associated with the risk of obesity and found that “two types of multilocus genetic risk scores were constructed to estimate the combined effect of selected SNPs” [10]. Nevertheless, it has not been clarified how much these SNPs contribute to obesity risk and related quantitative factors if combined and whether they can be considered as possible predictors of obesity.

Similarly, precision public health approaches can potentially enhance our understanding of the interplay between individuals and macrolevel factors such as occupational or environmental exposures that influence populations’ health. It integrates precision and population-based strategies to provide “the right intervention to the right population at the right time” by examining the different subgroups of the population based on contextual variables such as geospatial risk modelling and cluster analyses to understand health conditions that arise in a population [5]. A key success of precision public health is its potential to provide a more accurate and better assessment of characterized populations by combining big data and advanced omics technology on disease patterns, pathogens, exposures, behaviors, and susceptibility.

Today, the so-called omics technology is an innovative approach that promises to reform our understanding of the mechanisms explaining the complex biology behind obesity by collecting data on genes, metabolites, and examining biodatasets using genomics, epigenomics, transcriptomics, proteomics, metabolomics, and microbiomics methodological designs [11]. It has been increasingly successful in detecting common genetic susceptibility variants. For instance, to date, genome-wide association studies (GWAS) have identified more than 40 genetic variants associated with obesity and fat distribution [10–12]. Furthermore, efforts are being made to integrate different omics biomarkers into multi-omics approaches to explore and refine the characterization of phenotypes and serve as targeted precision prevention.

Consequently, screening specific biomarkers or genetic sequences in nonsymptomatic individuals can improve the current status quo of treatment of disease and will enable personalized treatment and therapy for obese individuals. When applied to obesity, integrating multi-omics approaches data analysis will enable a paradigm shift from the “one size fits all” approach towards precision obesity management, i.e. (1) precision prevention of the onset of obesity, (2) precision medicine and tailored treatment of obesity, and (3) precision risk reduction and prevention of secondary diseases related to obesity [13, 14]. Lastly, precision obesity management can potentially provide an accurate and better assessment of individuals with or without comorbidities. It allows to co-design a cost-effective and sustainable person-centered plan, and thus improves the quality of life in every individual with obesity and related comorbidities.

Despite the abundant literature, there are no diagnostic molecular markers which are suitable for risk prediction or the reduction of obesity development at an early stage. This knowledge gap can be closed by multi-omics approaches. In this paper, a targeted review was conducted to provide an overview of the different types of omics data: genomics, epigenomics, transcriptomics, proteomics, metabolomics and microbiomics for a better understanding of the etiology and pathophysiology of obesity development and how multi-omics approaches can be implemented in precision obesity management.

**Fig. 1:**
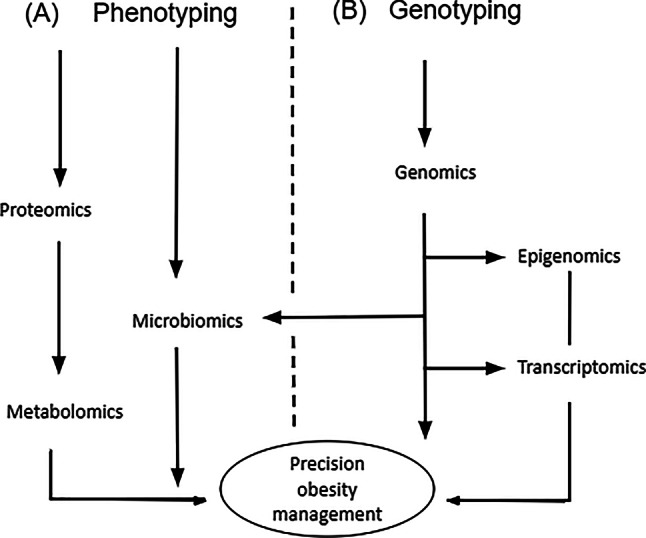
The various omics technologies applied to study obesity development

The different types of omics designs are grouped into two categories, the genome approach and the phenotype approach. Figure 1 provides a simple illustration, highlighting the two profiling strategies to integrate a multi-omics approach for precision obesity management.

In the following paragraphs, we describe the respective omics platforms, the definition and which technologies are used to detect them. Then we present the relation of the omics features with obesity and discuss the possibilities for applying it in the prevention, treatment, and risk reduction for secondary diseases.

## Genotyping

Genotyping is a clinical procedure that examines “an individual DNA sequence to determine a difference in the genetic mark-up of a genotype” [15]. Using gene expression profiling, it aims to predict and determine an individuals’ genetic variation and risk profiles for various diseases. In the last two decades, the application of genotyping strategies in clinical research have dramatically changed the pace of detection of common genetic susceptibility variants to chronic disease development. Furthermore, the development of DNA microarray-based techniques and next generation sequencing (NGS) analysis have enabled analyses of population-specific genetic traits [16]. Current methods of genotyping applications include genomics (DNA), epigenomics (DNA methylation) and transcriptomics (RNA).

### Genomics

Genomics is the most mature of omics technologies and focusses on identifying genetic variants associated with disease and prognosis [15]. While diet and sedentary lifestyles are predominately thought of as an obvious explanation for the rise of obesity, genome studies demonstrate the important role genetics play in the development of obesity. Understanding individuals’ phenotypes can potentially lead to targeted therapy to curb the obesity epidemic.

The first gene discovered associated with obesity was fat mass and obesity (*FTO*). Evidence has shown the role of* FTO* in food intake and its primary effects on diabetes mellitus and obesity [17–19]. A large-scale GWAS study among people of European descent has confirmed the strong association of the *FTO* locus with increased BMI and metabolic syndrome susceptibility [20]. The discovery of *FTO* has also revealed another strongly associated locus, the melanocortin 4 receptor gene (*MC4R*), which is associated with weight, fat mass and obesity [19, 21]. The *MC4R* gene was identified as the leading contributor of monogenic obesity and the interaction of *FTO* and *MC4R *genes on certain pathways are associated with obesity-related phenotypes.

Previous genome studies have also provided an insight into gender-specific differences in fat distribution, body shape, and susceptibility to obesity development. Evidence indicates that fat distribution is controlled by genetic factors as genes are involved in energy homeostasis, therefore affecting energy expenditure and energy intake through regulation mechanisms [12, 22, 23]. The studies revealed numerous genes harboring waist to hip ratio (WHR) loci, which regulate the fat distribution and fat depots. Eating behavior can have a direct and often unrecognized indirect effect on WHR loci, as the obesity susceptibility genes are highly expressed in the central nervous system, which are likely to influence appetite and satiety. Interestingly, one of the studies [23] found 14 WHR loci in women and only 6 loci in men, which were associated with an increase in waist circumference (WC) and BMI. The loci increasing the WHR in women demonstrate that gene variants can have gender-specific effects on the development of obesity and individual patterns of body fat.

Furthermore, family history was previously hypothesized as a good proxy for the genetic risk of developing obesity because of the shared environmental factors. This claim has been supported by several twin studies, which have estimated a 46–72% genetic heritability of BMI in children and adults [24, 25]; however, despite their success family studies were reliant on referral biases and were conducted in a small sample set to offer analytically confident detection of genetic variants involved in the regulation of obesity and related traits.

Consequently, over the last two decades, efforts have been made to identify genetic variants among predisposed obese individuals in a large sample set. With the emergence of GWAS, hundreds of genetic variants involved in different biological pathways have been associated with polygenic obesity. For instance, a GWAS with a sample size of 339,224 [26] and 244,459 [27] individuals, respectively, identified more than 97 BMI and 49 WHR obesity-associated loci. Another larger scale meta-analysis including 700,000 individuals yielded over 941 near-independent single nucleotide polymorphisms (SNPs) associated with BMI [28]; however, both studies indicated a limitation that a risk locus with > 5% effects for statistical significance were not detectable, which could equally contribute to understanding obesity risk and its traits.

There remains a gap in GWAS studies that obesity-related risk loci with small statistical significance are not detected in the analysis. To address this gap, a new approach has been developed known as genetic risk scores (GRS), which aggregates multilocus genetic risk profiles into a single predictive measure of obesity susceptibility [29]. Going forward, advances in the understanding of GWAS and the development of computational algorithms for GRS could facilitate targeted strategies for obesity prevention in early childhood. Lastly, GRS could be helpful in guiding lifestyle interventions targeted at high-risk individuals as these individuals at elevated genetic risk are also susceptible to secondary chronic diseases.

### Epigenomics

Epigenomics is a study of heritable changes and chemical modification. The epigenome consists of chemical compounds and DNA-associated proteins which may affect gene function without altering the DNA sequence [19]. There are many types of epigenetic markers including DNA methylation, histone acetylation, and non-coding RNAs (e.g., miRNAs), and they mediate a biological process called imprinting [30]. Failure in imprinting can cause the development of chronic disease and imprinting disorders are often clinical characteristics of obesity. Currently, the common method of examining epigenetic markers includes whole-genome bisulphite sequencing and epigenetic epigenomic array-based technologies.

It is hypothesized that epigenetic modification can occur early in life due to environmental influences, thereby permanently affecting metabolism and chronic disease risk later in life. A recent epigenomic study examined the effect of malnutrition during pregnancy on DNA methylation on the offspring [31]. The authors concluded that exposure to stress, undernutrition or overnutrition during gestation can “affect DNA methylation, histone post-translational modification as well as gene expression involved in insulin signalling and fatty acid metabolism”. The study suggests that the offspring will be predisposed to obesity and obesity-related secondary diseases. This claim is further corroborated by the Dutch Hunger winter study [32]. This human study examined the influence of maternal diet on DNA methylation in the offspring during gestation. The study showed maternal dietary changes caused permanent phenotypic variation and epigenomic alteration in the offspring, although the consequences of these variations are unexplored and remain unknown. Despite the limited human study on this subject, early detection of epigenetic changes can be used as early indicators of disease status for metabolic syndrome and cardiovascular disease in low-risk individuals.

Further studies in epigenome-wide association studies (EWASs) have also provided new lines of evidence identifying epigenetic modifications correlating with diseases in a larger study population. For instance, in the REGICOR study based on a sample of 641 participants and a sample of 2515 participants in the Framingham Offspring cohort, 70 CpG regions were associated with BMI [33]. The authors investigated the epigenetic effect of CpG loci in obesity development and found that these biomarkers explained 26% and 29% of the variability of BMI and waist circumference, respectively. This suggests that identifying an expressed CpG locus in nonsymptomatic individuals can be a potential diagnostic marker of obesity and risk for metabolic disorders.

Despite the challenge in the interpretation of epidemiological data, many studies have demonstrated the importance of epigenetic modifications and how environmental factors can influence behavioral changes and exert increased obesity risk later in life. Epigenomic modification can occur due to genetic, tissue differentiation, or exposure to environmental factors and behavioral changes. For instance, lifestyle and environmental factors “such as smoking, unbalanced diet, and infectious disease can expose a person, which prompts a chemical response, which in turn often lead to changes in the epigenome” [30]. Further EWAS studies can contribute to understanding pathways involved in the pathogenesis of obesity and identifying genes influenced by epigenetic biomarkers. Therefore, novel knowledge on epigenomic biomarkers and mechanisms can contribute to identifying potential therapeutic biological targets for precision obesity management and thus providing tailored lifestyle interventions.

### Transcriptomics

Transcriptomics technology examines transcriptome organisms, which are made up of Messenger RNAs (mRNAs), non-coding RNAs (ncRNAs), and small RNAs (sRNAs). Studying the transcriptome is crucial because it contains information about how each cell type functions under normal development in contrast to how they function in pathological conditions [34]. Next generation sequencing (NGS) and RNA sequencing (RNA-seq) are commonly used technologies to collect transcriptome profiling.

With the arrival of NGS, transcriptome profiling and microarrays transcriptional profiling have become the most common approaches for investigating chronic diseases on a molecular level and gene expression analysis [34]. For instance, tissue-specific analyses of the mRNA transcriptome of adipocytes showed that more than 1000 expressed genes were altered in obese subjects as compared to lean individuals [35]. Adipose tissue is the largest organ that plays a crucial role in regulating energy homeostasis and adipocytes serve as a “reservoir for energy storage and utilisation” [36]. Therefore, an increased number of adipocytes among obese individuals indicates dysfunction in white adipose tissue (WAT) and its obesity-associated metabolic complications such as insulin resistance (IR).

In another study [37] the authors carried out transcriptional profiling of peripheral blood among obese and lean subjects to investigate the biological processes related to the regulation of body mass. The study found an increased transcript level in genes involved in ribosome, apoptosis and oxidative phosphorylation pathways among obese subjects, which are consistent with an altered metabolic profile including increased protein synthesis, and increased energy demands. Although a dietary lifestyle intervention can be adequate to manage metabolic alterations, understanding the changing expression levels of each transcript and their effect on metabolic modification represents an opportunity for a therapeutic target for precision obesity management.

Another important discovery in transcriptomic biomarkers related to obesity risk and its associated comorbidities is the circulating micro RNAs (cmiRNAs). These are released into the bloodstream and cerebrospinal fluid and serve as key messengers between cells and tissues [29]. A recent study identified 33 cmiRNAs with dysregulated expression in serum or plasma among obese subjects and many of the genes identified are involved in fatty acid metabolism and phosphoinositide 3‑kinase (PI3K-Akt) pathways [38]. The study highlights the regulatory role of miRNAs in adipose tissue and the potential for diagnostic and therapeutic intervention of obesity and related secondary diseases.

Furthermore, transcriptomics studies have examined the role of miRNAs in obesity to better understand their regulatory roles in adipogenesis, adipocyte differentiation, and insulin signalling. Most human protein-coding genes are regulated by at least one miRNA and specific miRNA signatures have been described in many diseases, including obesity, type 2 diabetes mellitus, and cardiovascular diseases (CVD). Sequencing technology has allowed the identification of non-coding RNAs (ncRNAs), such as miRNAs and long ncRNAs (lncRNAs), and a greater understanding of transcriptional regulation [29]. Two transcriptomics studies [39, 40] have noted that miRNAs elicit posttranscriptional repression of gene expression and therefore specific miRNAs were differentially expressed in adipose tissue of obese individuals as compared to those with normal weight. Although these study findings are based on a limited sample set, they provide valuable insights into the underlying mechanisms of the progressive energy imbalance and fat distribution observed during obesity progression.

## Phenotyping

Phenotypes refer to the “physical appearance, biochemical characteristics and physiological function of an individual organism” which are largely influenced by the interactions between genetic effects and environmental factors such as what one eats, how much one exercises, and how much one smokes [17]. Following that, phenotyping is the study and evaluation of the phenotypes of a given genotype [41]. Phenotyping technology enables a simultaneous collection of transcription, protein, and metabolic modification in response to the environment [42]. To better understand the underlying mechanism of obesity by metabolic pathways, phenotyping methods can be fundamental. The most common methods for molecular composition analysis are nuclear magnetic resonance spectroscopy (NMRS) and mass spectrometry (MS).

### Metabolomics

Metabolites are small molecules that are involved in metabolic pathways, which are often “impacted by genetic variation, epigenetic status, enzyme activity, and environmental factors” [19]. For instance, an accumulation of metabolites can result in reduced enzyme activity that is exacerbated by lifestyle choices such as minimal physical activity, and nutrient intake which can have a significant impact on the metabolomic profile of an individual [41]. Metabolites are commonly measured in tissue samples or body fluids such as blood or urine.

Metabolomics is therefore a widely used analytical technique that measures changes in the metabolite profile within a cell, tissue, or organism [42]. It analyzes small molecule types such as lipids, carbohydrates, amino acids, fatty acids, or organic acids of cellular metabolic functions. In the past, metabolomics has been used for numerous discoveries of clinical conditions and these novel biomarkers have provided a better understanding of disease progression and metabolic pathways [43].

There are six metabolic pathways involved to better understand and predict the risk of obesity development. These are glucose metabolism and the citric acid cycle, lipid/fatty acid metabolism, bile acid metabolism, choline metabolism, amino acid metabolism, and creatine metabolism [44, 45]. The amino acid metabolism is the most important physiological phenomenon, and studies indicate that disorders in amino acid metabolism are related to the occurrence of insulin resistance and other metabolic conditions. Similar studies among obese children [46] and young Chinese men [47] show that obese subjects have higher concentrations of branched-chain amino acids (BCAAs) and, consequently, the products of their degradation. This suggests that an accurate assessment and prediction of insulin resistance and obesity risk factors can be determined by amino acid profiles.

Data from a study conducted among obese Japanese individuals found an increased level of the eight amino acids alanine, arginine, asparagine, glutamine, leucine, tyrosine, valine, and phenylalanine compared with lean subjects [48]. Similar findings were reported in another study conducted in Finland [49]. The authors concluded that “BCAAs, aromatic amino acids (AAAs), and orosomucoid” are major risk factors for the onset of obesity. The study also found an increased level in four acylcarnitine species (C3, C5, C6, and C8:1) in obese subjects. An increased level of plasma levels of BCAA in obese individuals reflects changes in metabolic signature and obesity deregulation.

Further metabolic pathway analysis in fatty acid biosynthesis, phenylalanine metabolism, leucine, and valine degradation can help distinguish the difference between metabolically healthy obese (MHO) individuals and metabolically unhealthy obese individuals. Although both groups meet the traditional BMI criteria for obesity, one consistent criterion to distinguish the two is based on abnormal metabolic phenotypes [19]. A Taiwanese study [50] conducted in a weight loss clinic to identify a potential difference in the metabolic profile found that there was an alteration of serum metabolites including L‑kynurenine, glycerophosphocholine (GPC), glycerol 1‑phosphate, glycolic acid, tagatose, methyl palmitate, and uric acid between obese metabolically healthy and unhealthy subjects. An increased level of non-essential fatty acids including oleic acid, palmitic acid, palmitoleic acid, stearic acid, stearoyl carnitine, 2‑hydroxybutanoic acid, and 3‑hydroxybutanoic acid was also reported in metabolically healthy obese individuals, which are associated with dyslipidemia, an imbalance of lipids. Similar studies are crucial in the future to predict the onset of metabolic, and cardiovascular risk among MHO. Moreover, an accurate identification of individuals with these conditions can lead to appropriate and precision treatment.

Lastly, the role of carbohydrates in obesity development is also an important study to understand energy imbalance and the accumulation of fat depots in obese individuals. The effects of dietary composition, particularly excessive consumption of high-fat and high-calorie diets are traditionally associated with obesity. One explanation is that the increase in energy intake can disrupt energy balance and can lead to a metabolic syndrome such as loss of glycemic control, dyslipidemia, hypertension and obesity [51]. Several interventional studies have demonstrated the beneficial effects of dietary fibers, particularly a low carbohydrate diet on body weight, food intake, glucose homeostasis and consequently the maintenance of weight loss [52, 53]; however, despite their short-term success, for an effective long-term precision weight management, further metabolic studies on the impact of genetic and epigenetic risk factors on the metabolome should provide further insight into how genes impact the metabolism and contribute to obesity development.

### Microbiomics

Metagenomics is a sophisticated sequencing technology that examines the microbial communities in the human body. The most common sequencing, an analytical technique to identify microbiome is 16S rRNA sequencing, which can accurately provide a complete picture of microorganisms such as bacteria, fungi, parasites, and viruses found in the human body.

Individuals are born with a “unique network of microbiota that is determined by their DNA” [54]. Most of the human microbiota, primarily bacteria are found in the gut, and evidence suggests that the human gut microbiota has a “significant impact on maintaining immune and metabolic homeostasis and protecting against pathogens” [55]. Studying the microbiomics status of the gut allows determination of the microbial diversity and how they change over time. Environmental exposure and diet can change one’s microbiome which can either be beneficial or potentially put them at a greater risk for chronic diseases. For instance, a disruption in microbial composition can lead to bowl diseases, a variety of neurological diseases, cardiovascular disorders, and respiratory diseases.

One possible explanation for reduced microbial diversity in the human gut is exposure to antibiotics. A recent meta-analysis reported a significant dose-response relationship between antibiotics exposure in very early life and childhood adiposity, showing elevated risk with repeated doses [56]. The study findings are supported by other epidemiological studies that claim that antibiotics exposure in the first year of life is associated with an increased risk of obesity during childhood and later in life [57, 58].

An important discovery in microbiome studies is the significant effect of dietary changes in bacterial metabolism especially in short-chain fatty acids (SCFA) and amino acids, which play an important role in “metabolic modulation, appetite regulation, and immune function” [17]. Data findings from a recent study [59] show a higher concentration of acetate in blood and feces, propionate and valerate in feces, and butyrate in feces produced by fermentation of dietary fiber in the gut were found in obese individuals compared with lean subjects. It is an important study because the *Bacteroidetes *phylum produces acetate and propionate while the *Firmicutes *phylum mainly produces butyrate for primary metabolic function. Although the study findings do not provide information on the causes for dysbiosis, an imbalance within the microbiome, the study highlights the link between host microbiota, digestion, and metabolism.

Microbiome studies that examine the proportion of the *Bacteroidetes *and *Firmicutes* phyla between obese and lean subjects show the correlation between the microbiome and fat deposition. Several data show that there is a significantly reduced level in the abundance of *Bacteroidetes *and an increased level in the* Firmicutes* phyla in obese subjects [60–62]. The *Bacteroidetes *and *Firmicutes *are the two largest beneficial bacteria found in the human gut, which “together comprise 90% of the microbiota of the adult gut” and they play an important role in human metabolism and energy homeostasis [55]; however, other subsequent studies found no difference between obese and lean groups in the proportion of *Bacteroidetes*/*Firmicutes *and its relation to obesity development [63–65]. The discrepancy in the data illustrates that the influence of the gut microbiome on obesity is complex and multifactorial.

Nevertheless, the relationship between the microbiome and obesity is best illustrated in weight loss interventional studies [52, 53, 66]. In one of the studies [66] participants were placed on either fat-restricted diet or a carbohydrate-restricted, low-calorie diet to monitor their fecal microbiota for 1 year. The study found an increased abundance of *Bacteroidetes* as their body weight decreased, slowly transitioning from the signature obese group to the lean group. This suggests that a modification in the gut microbiome is a personalized therapeutic option for weight-loss interventions for obese individuals. Although excessive consumption of high-fat and high-calorie diets and minimal physical activity can result in an energy imbalance, which subsequently leads to a metabolic syndrome, future studies on the microbiomics status of the gut are necessary to gain valuable information into how genes impact metabolism in obesity development [19]. Moreover, the relationship between the composition of the microbiome and an individual’s lifestyle choices together with the effects that they have on the metabolism are crucial steps for future studies to be able to translate this into precision obesity prevention.

### Proteomics

Proteomics is the study of large-scale analysis and quantification of proteins present in biological samples consisting of cells or tissue [67]. It is an important area of research to examine the characterization of the proteome and to better understand the mechanism of diseases. For instance, secreted proteins constitute an important class of molecules expressed by approximately 10% of the human genome, therefore, the serum/plasma proteome provides a useful resource for monitoring molecular events of pathological changes that occur in obesity [68]. With the development of MS technology, proteomics has enabled the identification and biochemical characterization of all the proteins in a cell that are associated with obesity and its comorbidities. Furthermore, the proteomics approach can detect posttranslational protein modifications and protein interactions that cannot be detected by genomics and transcriptomics.

Previous population-based proteomic studies in obesity have been based on small samples and had limited analytical and outcome reproducibility [69, 70]. Since then, well-established technologies such as matrix-assisted laser desorption/ionization coupled with time-of-flight mass spectrometry (MALDI-TOF-MS), liquid chromatography coupled with electrospray ionization mass spectrometry (LC-ESI-MS), surface-enhanced laser desorption/ionization TOF mass spectrometry (SELDI-TOF-MS), and protein microarray have been improved in large sample analyses in areas from protein separation to protein identification [71].

The characterization of protein expression in organs during obesity can help unveil the biological impact of obesity and a greater understanding of the metabolic syndrome. Using the latest technology in MS analysis, a study [72] by Jové et al. investigated the protein differences in omental and subcutaneous adipose tissue, a classification of white adipose tissue of healthy obese individuals. The authors identified 43 differentially expressed proteins including those that have been linked “to lipid and glucose metabolism, lipid transport, protein synthesis, and folding inflammation and the cellular stress response” which have an association with metabolic traits in body mass index and insulin sensitivity. Subcutaneous adipose tissue is a “fat found under the skin and largely around the hips, thighs, and buttocks” and omental adipose tissue is a type of visceral adipose tissue (central obesity), which is an excess accumulation of fat in the abdominal region and is commonly associated with metabolic dysfunction [19]. Although the underlying mechanism for this association remains unclear, adipose tissue exhibits a location-specific lipid profile that may contribute to obesity and several metabolic risk factors.

A subsequent study compared the plasma proteomes of two large independent cohorts of obese patients in Canada and Europe among 1002 obese individuals using shotgun MS-based proteomic measurements [73]. The study found statistically significant associations with BMI for the following biomarkers: complement factor B (CFAB), complement factor H (CFAH), complement factor I (CFAI), C‑reactive protein (CRP), proline-rich acidic protein 1 (PRAP1), and the calprotectin complex formed by proteins S100-A8 and S100-A9. Among these proteins, CRP showed the strongest association with BMI, which has also been associated with clinical parameters linked with obesity and PRAP1 showed a strong positive association with fasting insulin levels [74]. Altogether, these findings suggest that chronic inflammation in obese persons could represent the underlying reason for the associations of these biomarkers with obesity.

### Integration of multi-omics biomarkers for precision obesity management

For many years, lifestyle intervention studies have primarily focused on physical activity and diets, which despite the maintenance of weight loss are a short-term success and often fall short. Although physical activity and healthy isocaloric nutrition are important tools in the prevention of obesity, treatment, and risk reduction for secondary diseases, precision obesity management requires a novel approach to move towards a sustainable lifestyle change.

In a multi-omics approach, the different methodologies are combined to examine the effect of the genome, epigenome, transcriptome, metabolites, microbiomes, and proteomes in the human organism, to provide insights into the underlying mechanisms of obesity development. Therefore, the different types of omics biomarkers “investigate changes at different molecular levels, examine the interplay between the molecules and the roles of different factors involved in the metabolic health deterioration” [17]. In other words, it allows better tracking of individual biological parameters that subsequently result in obesity, allowing the further depiction of the causal pathway to obesity. When applied to obesity prevention and management, each omics type could potentially help to detect specific biomarkers in people with risk profiles and perhaps guide healthcare professionals and decision makers develop individualized treatment plans according to the needs of the individual before the onset of obesity.

**Fig. 2:**
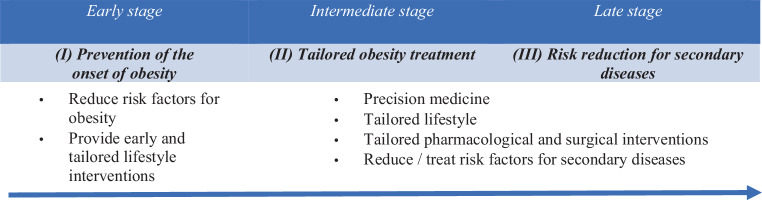
The three stages of precision obesity prevention management with the application of a multi-omics approach

There are several benefits of adopting multi-omics technologies in clinical and nonclinical settings, prognosis, and obesity prevention. First, multi-omics technologies can identify the changes in body composition that precede the onset of obesity to implement response to treatment and future patient prognosis. Second, multi-omics technologies can distinguish obese individuals that are phenotypically (and genotypically) heterogeneous. Third, novel therapeutic approaches and tailored weight-loss management can be implemented that are in response to the new biological drivers and particular obesity phenotypes. Figure 2 depicts a simple illustration of how multi-omics technologies can be implemented in obesity precision prevention.

In the early stage, multi-omics can be applied to support and guide nonsymptomatic and healthy individuals through various stages of their lives to prevent the development of obesity and its comorbidities. This can be achieved through the collection of routine clinical data, fitness data including data from spiroergometry and strength tests, data regarding nutrition, physical activity, mental health resources, distress, social capital, health literacy, quality of life, data about body composition, and blood findings. Moreover, additional population-wide biodata in intestinal microbiota, genetics and genomics as well as metabolomics, and a transferable minimum dataset can facilitate early-stage obesity prediction. Therefore, with the assistance of multi-omics technology, healthcare will be better equipped to address specific health issues and inequalities in subpopulations and effectively address the obesity epidemic. Moreover, incorporating multi-omics is a critical step for healthcare professionals, and policy makers to move towards implementing lifestyle interventions that will be balanced between health promotion and disease prevention, and thus this will subsequently contribute to maintaining and improving health while ensuring affordability.

During the intermediate stage, the focus lies on the treatment of obesity and examination of other factors such as lifestyle choices, environmental and societal factors that may have contributed to the development of obesity. Therefore, a personalized medicine can be implemented as an effective strategy to support people or groups with similar genetic or metabolic blueprints by tailored interventions. One of the aims of precision medicine is optimizing patient experiences. With the support of multi-omics, to create individualized tailored programs, the rebalancing between weight management and disease prevention can empower citizens over their own health, which can improve participation in health promotion programs. Moreover, tailored lifestyle interventions and long-term weight-loss strategies can be useful tools as well as tailored drug therapies and surgical interventions at the aggregate level.

At the third stage, the multi-omics approach can be applied for the risk reduction of secondary diseases. Advanced bioinformatics methods are especially advantageous to obtain a better understanding of the complexity and interactions of the biological systems that predispose obesity among individuals and to investigate clusters that exist in the subgroups of obesity. The aim would be to generate big data, based on individual data sets to draw conclusions on which social determinants can be addressed through the multi-omics approach. Furthermore, the multi-omics approach can support healthcare professionals and decision makers better address the needs of the individuals and the populations at large.

### Limitations of the omics approach

In practice, lifestyle interventions can equally be an important tool for prevention of the onset of obesity and risk reduction for secondary diseases. Together with multi-omics technology, they can help us to identify and predict individuals with susceptible gene and obesity risk profiles and guide us to provide a long-term intervention that is sustainable and effective.

However, for effective lifestyle interventions in precision obesity management, further work is needed in the application of the multi-omics approach before it can be implemented into a concrete intervention. At the moment, the transformation and interpretation of epidemiological data into accurate prediction and prognosis for obesity risk factors remain challenging. Furthermore, the inconsistent and conflicting results of epidemiological studies raise uncertainties about the validity of study designs, sample collection, measurement, and data analysis. The small number of participants in genome studies is another limitation that needs to be addressed in future research. Nevertheless, to address the issues of heterogeneity in sample size, national and international collaborative research networks can be developed to analyze larger sample sizes. Moreover, the issues of the interpretation of omics data can be addressed by increasing the demand for larger prospective cohort studies to validate findings and determine biomarker reproducibility before interventions are implemented as a public health strategy. Finally, further genome studies are needed to develop analytical infrastructures that can generate, analyze, and interpret multi-omics data as a basis for guiding precision obesity prevention strategies.

## Conclusion

Obesity is a global public health concern and the need for precision obesity prevention strategies is now. Fortunately, technological advancement can provide insights into the underlying mechanisms involved in obesity development. Multi-omics approaches can help to detect specific biomarkers in people with risk profiles and perhaps guide healthcare professionals and decision makers to develop individualized treatment plans according to the needs of the individual before the onset of obesity. Understanding the role of genetic and epigenetic factors and their influence in the transcriptome, proteome, metabolome, and microbiome is a critical step towards implementing a lifestyle intervention that is sustainable and effective. Furthermore, identifying an individual’s susceptibility to obesity will be a paradigm shift from a one-size-fits-all approach to an individualized care package, which is an important determinant for the success of interventions. Therefore, prevention programs can be developed to promote overall health and prevent obesity in the appropriate communities.

However, we must acknowledge that the transformation of large and heterogeneous omics data into biological knowledge and clinical parameters has proven challenging especially when different GWAS studies yield inconsistent results. Moreover, a consistent, statistical validation of biological samples must be the focus of further research. As such, we must continue to push forward in research initiatives that concentrate on addressing the limitations of omics data and provide guidelines for precision obesity management strategies.
